# Dental Deterioration

**Published:** 1904-09-24

**Authors:** 


### DENTAL DETERIORATION.

Dr. J. Sim Wallace, in a paper read before the British Dental Association, argues that there has been a progressive deterioration in the teeth of human beings. Dental caries, he says, was once almost unknown, whereas at the present day, in England at least, it is the most prevalent of all diseases. For the most part the development of caries in children takes place before the school age, and hence the dental inspection of children attending elementary schools, whilst a very desirable measure, would not fully meet the case. To prevent the increase of caries and the evil consequences which follow it, more attention, according to Dr. Wallace, must be paid to the nature of the food substances allowed in early life. The prolongation of the period of pap feeding, and the consequent absence of demand for the use of the muscles of mastication, cause imperfect development of the jaws and their related parts. Children should be encouraged from a very early date in the use of foods which make masticatory efforts essential, and this necessity should be explained to the mothers of young children. The use of soft, highly-prepared foods is largely responsible for the early development of dental caries. It will be remembered that to the same influence has been attributed the presence of adenoids in the naso-pharynx. Non-use of the muscles of mastication means a relatively inefficient blood supply to, and an imperfect development of, the upper portions of the alimentary canal. Discussions on these subjects are frequently repeated, and rival theories have their several innings with doubtless some measure of progress. But it is somewhat curious that physiology, which appears in some of its aspects to attain such definite results, is very far from having settled the various problems which present themselves in the subject of dietetics. When, if ever, that settlement is attained, many questions which at present perplex the physician, will have found satisfactory answers.

